# Clinical utility of small intestinal bacterial overgrowth (SIBO) testing in guiding management of gas-bloat symptoms after antireflux surgery

**DOI:** 10.1007/s00464-025-12092-7

**Published:** 2025-08-28

**Authors:** Naveed Chaudhry, Sven E. Eriksson, Inanc S. Sarici, Kelsi J. Swanson, Sarah Hanuschock, Ann M. DeWitt, Ping Zheng, Shahin Ayazi

**Affiliations:** 1 Foregut Division, Surgical Institute, Allegheny Health Network, 4815 Liberty Avenue, Suite 439, Pittsburgh, PA 15224 USA; 2 Chevalier Jackson Esophageal Research Center, Western Pennsylvania Hospital, Allegheny Health Network, Pittsburgh, PA USA; 3 Department of Surgery, Drexel University, Philadelphia, PA USA

**Keywords:** Small intestinal bacterial overgrowth (SIBO), Gas-bloat syndrome (GBS), Antireflux surgery, Antibiotic

## Abstract

**Background:**

Gas-bloat symptoms are common after antireflux surgery (ARS) and typically attributed to impaired gas venting from surgical changes. However, small intestinal bacterial overgrowth (SIBO), potentially linked to chronic PPI use, may also contribute. Despite this, the role of SIBO in post-ARS bloating remains underexplored. This study aimed to assess the utility of SIBO testing in patients with persistent gas-bloat symptoms after ARS.

**Methods:**

Patients with persistent bloating after primary ARS were offered SIBO breath testing and gastric emptying scintigraphy. Those with normal gastric emptying who completed testing were included. A gas-bloat score ≥ 4 on the GERD-HRQL questionnaire was considered severe. Outcomes were compared between SIBO-positive and SIBO-negative patients. SIBO-positive patients were treated with antibiotics and reassessed.

**Results:**

Among 71 patients with postoperative gas-bloat symptoms following ARS, 40 (56.3%) tested positive for SIBO. Compared to SIBO-negative patients, SIBO-positive patients had significantly higher postoperative gas-bloat scores (median [IQR] 4.0 [4.0–5.0] vs 3.0 [3.0–4.0], *p* < 0.001) and more frequent severe symptoms (77.5% vs 41.9%, *p* = 0.003), despite similar GERD-HRQL total scores, acid exposure, and satisfaction (*p* > 0.05). Following antibiotic therapy, severe gas-bloat symptoms improved markedly (77.5% to 23.1%, *p* < 0.001), with corresponding reductions in gas-bloat scores (4.0 [4.0–5.0] to 2.0 [1.0–3.0], *p* < 0.0001) and GERD-HRQL total scores (10.5 [7.0–19.0] to 5.0 [2.0–14.0], p = 0.002). After treatment, SIBO-positive patients had significantly better GERD-HRQL total scores than SIBO-negative patients (5.0 [2.0–14.0] vs 15.0 [7.0–26.0], *p* < 0.0001). Satisfaction also increased from 42.5% to 69.2% (*p* = 0.021). Post-treatment, SIBO-positive patients had significantly lower gas-bloat (*p* = 0.004), lower regurgitation scores (*p* = 0.003), and higher satisfaction (*p* = 0.015) than SIBO-negative patients.

**Conclusion:**

SIBO was present in over half of patients with persistent bloating after ARS and was associated with more severe symptoms. Antibiotic treatment led to substantial symptom improvement and increased satisfaction. These findings suggest that SIBO testing may guide targeted therapy and improve outcomes in selected patients following antireflux surgery.

**Graphical abstract:**

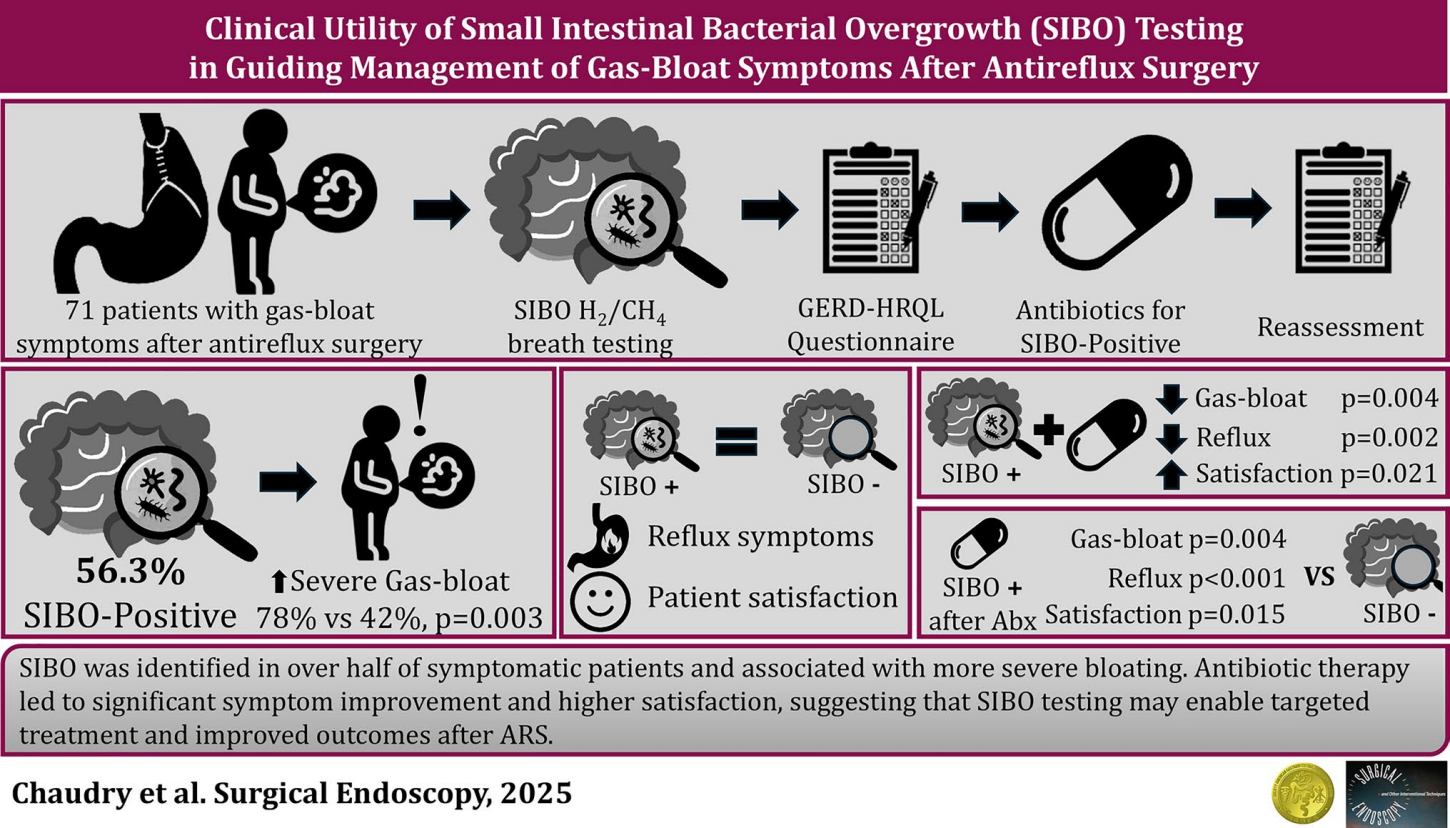

Antireflux surgery (ARS) is an effective treatment for patients with medically refractory gastroesophageal reflux disease (GERD), providing durable symptom relief and high rates of patient satisfaction. However, postoperative gas-bloat symptoms are a common source of dissatisfaction, even in the setting of excellent reflux control. These symptoms represent a heterogeneous constellation of complaints (abdominal bloating, early satiety, distension, nausea, and an impaired ability to belch or vomit), which are variably present and mechanistically complex. Despite excellent reflux control, gas-bloat symptoms occur in up to 40% of patients after ARS and often cause more postoperative discomfort than residual heartburn or dysphagia [1–3]. Proposed etiologies of gas-bloat symptoms include impaired gas venting through a reinforced lower esophageal sphincter (LES), vagal nerve injury, aerophagia, and gastroparesis [4]. When gastroparesis is excluded, postoperative gas-bloat is typically attributed to non-specific clinical labels or diagnoses of exclusion. Recent data, however, suggest that small intestinal bacterial overgrowth (SIBO) is an important and underrecognized contributor to gas-bloat symptoms, offering an objective diagnosis that can be readily assessed [5]. However, these studies have focused on SIBO before surgical intervention. There is a paucity of data on the relationship between SIBO and postoperative gas-bloat symptoms.

SIBO is defined by the presence of an excessive concentration of bacteria in the small intestine, resulting in excessive fermentation of carbohydrates and gas production. It has been linked to gastrointestinal dysmotility, chronic proton pump inhibitor (PPI) use, and altered intestinal microbiota, all factors prevalent among patients with GERD and those undergoing ARS [5–10]. The symptoms of SIBO are non-specific and often include abdominal bloating, distension, nausea, and changes in bowel habits, features that overlap clinical presentation of gas-bloat after ARS. Hydrogen-methane breath testing (HMBT), a noninvasive and cost-effective diagnostic tool, detects excessive exhaled gases that reflect small intestinal fermentation of oral substrates. Despite its utility, HMBT is not routinely employed in the assessment of gas-related symptoms after ARS [11].

Given this symptomatic overlap and the high prevalence of multiple predisposing factors in the ARS population, SIBO may represent an under recognized cause of postoperative gas-bloat that can be both objectively diagnosed and treated. However, clinical relevance of a positive HMBT and responsiveness to treatment of SIBO in the setting of gas-bloat symptoms after antireflux surgery remain poorly defined. The primary aim of this study was to evaluate the clinical utility of SIBO testing in patients with persistent gas-bloat symptoms after ARS. The secondary aim was to determine if targeted antibiotic therapy in patients with SIBO would improve both symptom burden and patient satisfaction after ARS.

## Methods

### Study population

Patients who underwent primary antireflux surgery at Allegheny Health Network hospitals (Pittsburgh, PA) over a 4-year period and who subsequently complained of persistent postoperative gas-bloat symptoms were evaluated. A cohort of patients were offered SIBO hydrogen and methane breath testing and gastric emptying scintigraphy. Patients who completed postoperative SIBO testing and had no evidence of delayed gastric emptying were included. Patients who did not complete postoperative questionnaires were excluded. Surgical outcomes were compared between the SIBO-positive and SIBO-negative patients after surgery. SIBO-positive patients were then treated with antibiotics and all patients were reassessed. This study was evaluated and approved by the Institutional Review Board of the Allegheny Health Network (IRB 2024–008-AHNMR).

### Clinical assessment

Patients underwent a complete preoperative clinical evaluation including upper endoscopy, videoesophagram, esophageal pH-monitoring, and high-resolution manometry. A DeMeester score < 14.7 on pH monitoring was considered normal. All patients were asked to complete the GERD health-related quality-of-life (GERD-HRQL) questionnaire preoperatively, at 6 months, at 12 months, and annually after surgery. The GERD-HRQL consists of 16 questions that specifically address GERD symptoms. Each question has a score ranging from 0 to 5, indicating “No symptoms,” “Noticeable but not bothersome symptoms,” “Occasionally bothersome symptoms,” “Daily bothersome symptoms,” “Symptoms that affect daily activities,” and “Symptoms that are incapacitating to daily activities,” respectively. Within the GERD-HRQL are specific items pertaining to the symptoms of heartburn, regurgitation, dysphagia, and gas-bloat. A GERD-HRQL total score greater than 30 was considered as severe reflux symptoms. Patients with a score ≥ 4 on the gas-bloat-specific question of the GERD-Health-Related Quality of Life (GERD-HRQL) questionnaire were considered to have severe gas-bloat symptoms. Patients who reported postoperative gas-bloat symptoms, but scored < 4 on the gas-bloat-specific question were said to have mild to moderate symptoms.

Endoscopy was performed pre- and postoperatively by experienced foregut surgeons to assess key anatomical features, including crural impression, gastroesophageal junction (GEJ) position, presence of esophagitis graded by Los Angeles classification, and whether the surgical repair was intact or there was anatomical failure (recurrent hiatal hernia, disruption or slippage of the fundoplication/MSA device). Anatomical failure was assessed annually using upper endoscopy for up to 5 years.

### Patient selection and surgical procedure

Following the subjective and objective foregut evaluation patients were risk stratified by their individual factors and presented with treatment options. A shared decision-making model was implemented taking into account all patient factors to arrive at the final management decision.

Nissen fundoplication was completed laparoscopically in all patients. The surgical technique involved: taking down the gastrohepatic ligament and continuing along on to the right crus to divide the peritoneum overlying the anterior surface of the esophagus. Further dissection was carried out over the left crus. Restoration of an intraabdominal esophageal length ≥ 3 cm was achieved with complete trans-mediastinal mobilization of the distal esophagus. The short and posterior gastric vessels were taken down and the fundus of the stomach was completely mobilized from the retroperitoneum. A floppy 360° fundoplication was created through the retroesophageal window. The hiatus was closed with posterior nonabsorbable stitches without pledgets or mesh. Anterior and posterior vagal nerve trunk were identified and protected in all patients.

The LINX device (Ethicon, Johnson & Johnson, Shoreview, MN) consists of a series of magnetic titanium beads. The beads are interlinked with independent titanium wires to form a flexible and expandable ring with a “Roman arch” configuration. Each bead can move independently of the adjacent beads, creating a dynamic implant without limiting the esophageal range of motion. The device is manufactured in different sizes, ranging from 13 to 17 beads, and is capable of nearly doubling its diameter when all beads are separated. MSA is performed laparoscopically and consists of complete posterior mediastinal esophageal mobilization with restoration of intra-abdominal esophageal length (≥ 3 cm), interrupted posterior crural closure (without pledgets or mesh), and device placement at the level of the GEJ with the posterior vagus nerve trunk located on the outside of the magnetic ring.

### SIBO testing

All patients underwent HMBT SIBO testing after presentation with postoperative gas-bloat symptoms. Patients consumed a standardized lactulose or glucose substrate and then provided breath samples at regular intervals for 2–3 h. Hydrogen-methane breath testing relies on a fermentation of the oral substrate by gut microbiota. Hydrogen and methane gases are produced by gut flora from the ingested substrates, particularly the colonic flora in patients with carbohydrate malabsorption and from small bowel bacteria in patients with SIBO. Eighty percent of the gases like hydrogen and methane are eliminated with the flatus and the remaining 20% are absorbed and exhaled by lungs, which can be measured in breath. In a Glucose-Hydrogen-Breath-Test (GHBT), the rise in hydrogen by 12 parts per million (ppm) above basal following administration of 50–100 g glucose due to bacterial fermentation of the substrate in small intestine was diagnostic of SIBO. In the presence of SIBO, two peaks were seen during a Lactulose-Hydrogen-Breath-Test (LHBT): the first one due to bacterial fermentation of lactulose in small bowel and the second one after lactulose reaches colon. Since the number of bacteria in colon is higher than that in the small bowel even in patients with SIBO, a rise in breath hydrogen more than 20 ppm above basal was diagnostic of SIBO.

### Gastric emptying scintigraphy

Gastric emptying scintigraphy was performed in patients with nonspecific symptoms common in GERD but potentially suggestive of gastroparesis (e.g. nausea, vomiting, and bloating). GES was performed at 1-h intervals for 4 h after ingestion of a standardized solid meal. A percent meal retention at 4 h of > 10% was considered delayed gastric emptying.

### Statistical analysis

Patients were divided into two groups based on the presence of postoperative SIBO testing. Demographic characteristics, preoperative clinical data, and objective findings were compared between the groups. Postoperative outcomes were assessed and compared at 1 year. Patients with SIBO were then treated with antibiotics and all patients were reassessed. Additionally, to determine the association between HMBT and gas-bloat symptom severity, the results of HMBT were compared between patients with severe (≥ 4) gas-bloat and mild to moderate (< 4) gas-bloat symptoms.

Continuous variables were expressed as mean with standard deviation (SD) or median with interquartile range (IQR). Categorical variables were expressed as frequency and percentage. Comparisons between groups were performed using Pearson’s chi-square test for categorical variables and Mann–Whitney U test for continuous variables. A *p*-value < 0.05 was considered statistically significant. All statistical analyses were conducted using SAS software (SAS Institute Inc., Cary, NC).

## Results

The final study population included 71 patients who presented with gas-bloat symptoms after primary antireflux surgery and who subsequently underwent SIBO breath testing. At a mean (SD) follow-up of 19.2 (21.2) months there was a significant improvement in the GERD-HRQL score from 34.0 (22–49) to 12.0 (7–22), *p* < 0.0001, with 62% of patients reporting severe gas-bloat symptoms and only 40.8% patient satisfaction. Freedom from PPIs was 82.9% and 81.8% achieved normal esophageal acid exposure.

Magnetic sphincter augmentation (MSA) was performed in 39.4% of patients. Patients who underwent MSA were significantly younger [55.5 (40–63) vs 62.0 (49–72), *p* = 0.028], but otherwise had no differences in preoperative GERD-HRQL total score, bloating score, or DeMeester score (p > 0.05 for all). After surgery this population of patients with postoperative gas-bloat symptoms had no difference in postoperative GERD-HRQL scores, patient satisfaction, PPI use, or pH-normalization between MSA and fundoplication (p > 0.05 for all). There was no difference in postoperative SIBO testing results between surgical procedures (p = 0.807).

### Comparison of preoperative characteristics between SIBO-positive and SIBO-negative patients

After primary antireflux surgery, 40 patients (56.3%) with gas-bloat symptoms tested positive for SIBO. Among SIBO-positive patients, 30 (78.9%) tested positive for Hydrogen, while 15 (39.5%) tested positive for Methane. The demographic and preoperative clinical characteristics of SIBO-positive and SIBO-negative patients are compared in Table 1. There were no significant differences between the two groups in age, gender, BMI, GERD-HRQL total score, or DeMeester scores (*p* > 0.05). Notably, the preoperative gas-bloat score was also comparable (*p* = 0.290).

**Table 1:** Comparison of demographic and baseline clinical characteristics between patients with SIBO positive and SIBO negative

Characteristics	SIBO negative (N = 31)	SIBO positive (N = 40)	p value
Age, y, median (IQR)	62 (47–67)	57 (46–68)	0.767
Sex (female), N (%)	26 (83.9%)	32 (80.0%)	0.763
BMI, median (IQR)	29 (26–32)	28 (25–32)	0.664
BMI > 30, N (%)	9 (29.0%)	12 (30.0%)	0.929
GERD-HRQL, median (IQR)			
Total score	37.0 (20.0–50.0)	30.0 (24.0–48.0)	0.463
Heartburn score	25.0 (15.0–33.0)	21.0 (14.0–31.0)	0.422
Regurgitation score	13.0 (8.0–18.0)	12.0 (5.0–20.0)	0.696
Dysphagia score	2.0 (0.0–3.0)	2.0 (0.0–3.0)	0.975
Gas bloat score	4.0 (3.0–5.0)	3.0 (4.0–5.0)	0.290
PPI use, N (%)	29 (93.5%)	38 (95.0%)	0.902
Hiatal hernia > 3 cm, N (%)	9 (29.0%)	13 (33.3%)	0.700
Esophagitis LA C/D, N (%)	4 (12.9%)	1 (2.6%)	0.166
DeMeester score, median (IQR)	32.1 (18.5–55.9)	33.8 (11.0–43.1)	0.546
Primary antireflux surgery			
Nissen fundoplication	18 (58.0%)	25 (62.5%)	0.807
LINX	13 (42.0%)	15 (37.5%)	

### Impact of SIBO on surgical outcomes

Surgical outcomes between SIBO-positive and SIBO-negative patients are compared in Table 2. Patients in the SIBO-positive group had significantly higher gas-bloat scores with a higher incidence of severe gas-bloat symptoms (77.5% vs 41.9%, *p* = 0.003). GERD-HRQL total scores and patient satisfaction were comparable. The rate of postoperative LA C/D esophagitis and the percent of patients who achieved pH normalization were also comparable. Anatomical failure occurred in 24.6% of patients who had gas-bloat symptoms following antireflux surgery; 31.7% of Nissen fundoplications and 14.3% of MSA (*p* = 0.155). There was no difference in failure or need for revisional surgery between SIBO groups.

**Table 2:** Comparison of postoperative 1-year clinical and objective outcome at the time of the SIBO testing

Characteristics	SIBO negative (N = 31)	SIBO positive (N = 40)	*p* value
GERD-HRQL, median (IQR)			
Total score	15.0 (7.0–26.0)	10.5 (7.0–19.0)	0.225
Heartburn score	2.0 (0.0–9.0)	0.0 (0.0–7.0)	0.670
Regurgitation score	4.0 (0.0–12.0)	0.0 (0.0–5.5)	0.093
Dysphagia score	2.0 (0.0–4.0)	1.0 (0.0–2.5)	0.091
Gas-bloat score	3.0 (3.0–4.0)	4.0 (4.0–5.0)	< 0.001
Freedom from PPI, N (%)	24 (80.0%)	34 (85.0%)	0.750
Patient satisfaction, N (%)	12 (38.7%)	17 (42.5%)	0.810
LAC/D esophagitis	0 (0%)	0 (0%)	1.000
pH normalization, N (%)	17 (73.9%)	27 (81.8%)	0.478
Anatomical failure, N (%)	7 (23.3%)	10 (15.4%)	1.000
Need for Revision, N (%)	4 (57.1%)	4 (40.0%)	0.637

SIBO breath test results are compared between patients with severe vs mild to moderate postoperative gas-bloat symptoms in Table 3. Severe gas-bloat symptoms were associated with higher levels of hydrogen and methane at all levels in the gastrointestinal tract (*p* < 0.01 for all).

**Table 3:** Comparison of SIBO breath test results between patients with mild-moderate vs severe gas-bloat

Characteristics	Mild-moderate gas-bloat (N = 27)	Severe gas-bloat (N = 44)	*p* value
Hydrogen (H_2_) level, ppm^3^, Median (IQR)			
Overall	5 (2–20)	22 (7–72)	0.010
Small intestine	8 (3–23)	24 (11–63)	0.001
Transition	5 (2–20)	19 (4–70)	0.011
Large intestine	10 (2–20)	34 (12–79)	0.009
Methane (CH_4_) level, ppm^3^, Median (IQR)			
Overall	2 (1–5)	7 (4–12)	0.001
Small intestine	5 (2–11)	11 (5–19)	0.002
Transition	4 (1–7)	9 (4–16)	0.006
Large intestine	5 (2–9)	11 (5–24)	0.001
Combined H_2_ and CH_4_ level, ppm^3^, Median (IQR)			
Overall	9 (4–33)	31 (11–82)	0.002
Small intestine	14 (9–30)	37 (17–92)	< 0.001
Transition	10 (5–26)	33 (13–80)	0.002
Large intestine	14 (6–42)	50 (18–90)	0.002

### Impact of SIBO treatment on outcomes

**Fig. 1:**
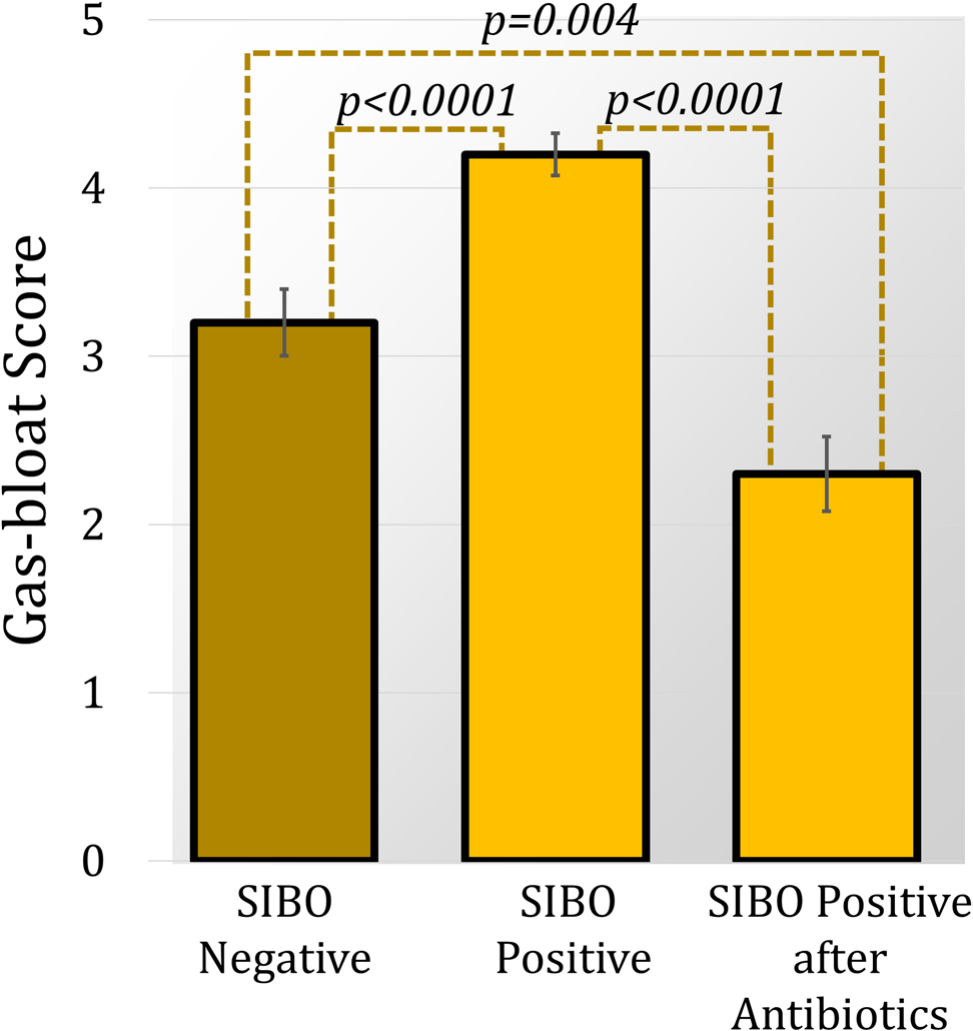
Comparison of mean (SE) gas-bloat scores between the SIBO-negative group and the SIBO-positive group before and after antibiotic treatment. After antireflux surgery SIBO-positive patients had significantly worse gas-bloat [4.2 (0.1) vs 3.2 (0.2), *p* < 0.0001]. However, after antibiotics the SIBO-positive group’s score significantly improved [4.2 (0.1) to 2.3 (0.2), *p* < 0.0001], significantly lower than the SIBO-negative group (*p* = 0.004)

**Fig. 2:**
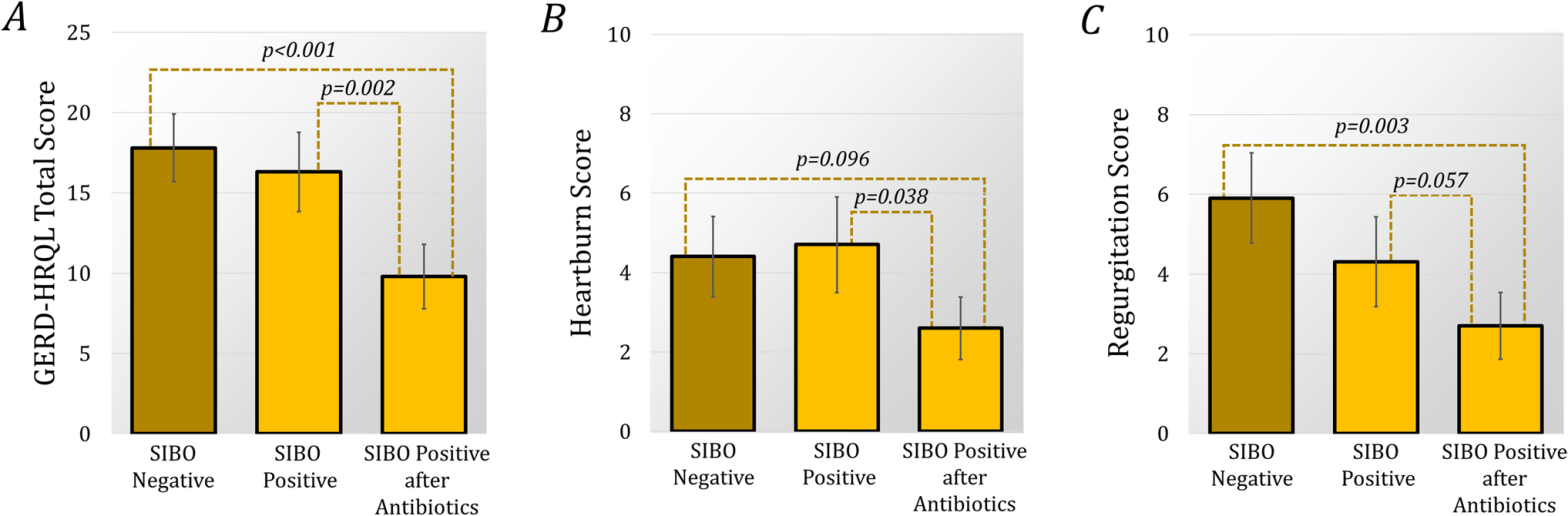
After antireflux surgery there was no difference in postoperative GERD-HRQL, heartburn, regurgitation or dysphagia scores between SIBO-positive and -negative groups (*p* > 0.05 for all). However, after SIBO patients were treated with antibiotics, there was a significant improvement in the mean (SE) (**A**) GERD-HRQL total score [16.3 (2.5) to 9.8 (2.0), *p* = 0.002] and (**B**) heartburn score [4.7 (1.2) to 2.6 (0.8), *p* = 0.038]. The (**C**) regurgitation score [4.3 (1.1) to 2.7 (0.8), *p* = 0.057] also improved, but dysphagia did not (*p* = 0.176). After treatment the SIBO-positive group had significantly lower GERD-HRQL total [9.8 (2.0) vs 17.8 (2.1), *p* < 0.001] and regurgitation scores [2.7 (0.8) vs 5.9 (1.1), *p* = 0.003]. The heartburn score was also lower [2.6 (0.8) vs 4.4 (1.0), *p* = 0.0096]

Among SIBO-positive patients, the most prescribed antibiotics were neomycin and rifaximin (77.5%), followed by cephalexin and metronidazole (12.8%), trimethoprim/sulfamethoxazole (5.1%), and tetracyclines (5.1%). Following treatment, gas-bloat symptoms significantly improved (Fig. 1). The prevalence of severe gas-bloat decreased from 77.5% to 23.1% (*p* < 0.001). Additionally, antibiotic treatment resulted in a significant improvement in the GERD-HRQL total score and heartburn score, with a trend toward improve in the regurgitation score (Fig. 2). The dysphagia score was unaffected by treatment. Patient satisfaction also significantly improved, but proton pump inhibitor use did not change significantly.

**Fig. 3:**
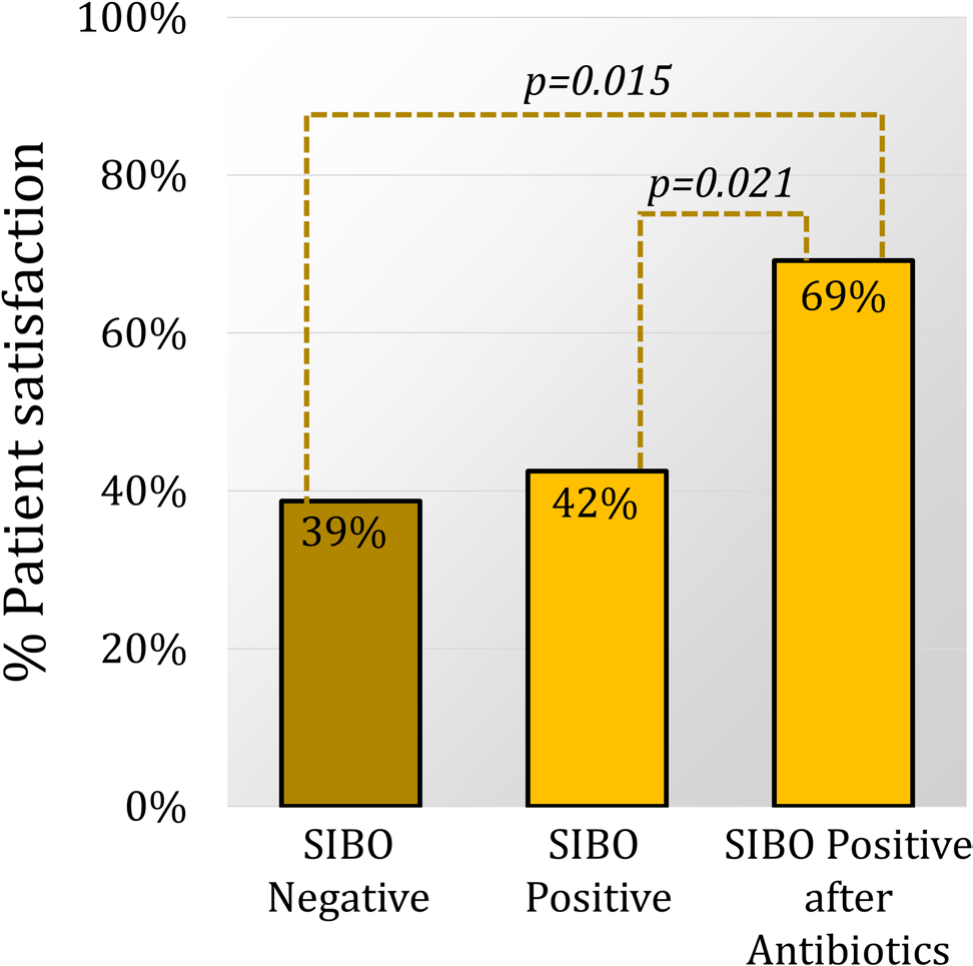
After antireflux surgery there was no difference in patient satisfaction between groups (*p* = 0.810). However, in the SIBO-positive group, treatment with antibiotics resulted in a significant improvement in patient satisfaction from 42 to 69%, *p* = 0.021. Compared to SIBO-negative patients, SIBO-positive patients who received antibiotics were significantly more satisfied with their surgical outcome (69% vs 39%, *p* = 0.015)

Before antibiotic treatment, SIBO-positive patients reported more severe gas-bloat symptoms than SIBO-negative patients. However, following antibiotic treatment this trend reversed, gas-bloat scores were significantly higher in the SIBO-negative group. Additionally, the post-antibiotic improvements in the GERD-HRQL total score and regurgitation score resulted in significantly lower scores than the SIBO-negative group, with an additional trend toward a lower heartburn score. Patient satisfaction was also significantly higher in the SIBO-positive group after antibiotic treatment than the SIBO-negative group (Fig. 3).

## Discussion

Antireflux surgery (ARS) is the most effective treatment for medically refractory gastroesophageal reflux disease (GERD), yet its utilization remains limited, in part due to concerns about postoperative side effects [1]. Among these, gas-bloat symptoms are particularly common and are often cited by patients as a reason for declining surgery, even when objective testing confirms a significant reflux burden [1]. When gastroparesis is absent, these symptoms are typically attributed to gas-bloat syndrome (GBS), a diagnosis of exclusion thought to result from impaired venting through a surgically augmented lower esophageal sphincter [12, 13]. Prior studies have shown that GBS substantially impacts patient satisfaction and perceived surgical success, yet therapeutic options remain largely palliative, aimed at controlling symptoms rather than addressing the root cause [4]. Small intestinal bacterial overgrowth (SIBO) has been identified as a potential etiology of bloating in GERD patients, particularly in the context of chronic acid suppression, which disrupts the gut microbiota and impairs colonization resistance [5, 6]. Given the symptomatic overlap between SIBO and GBS, and the high prevalence of PPI use in this population, it is plausible that a subset of patients diagnosed with GBS may, in fact, have undiagnosed SIBO. Their symptoms may therefore also respond to targeted antibiotic therapy. In this study, we evaluated patients with persistent postoperative gas-bloat symptoms for the presence of SIBO, compared their outcomes to those without SIBO, and reassessed symptoms after treatment. Our findings suggest that SIBO-positive patients represent a distinct clinical phenotype with more severe bloating, but one that is amenable to targeted therapy with clinically meaningful improvement in symptom burden and satisfaction after antibiotics.

In our population of patients with postoperative gas-bloat symptoms, SIBO-positive patients reported significantly more severe symptoms compared to SIBO-negative patients. Moreover, patients with severe symptoms had significantly higher production of both methane and hydrogen at all levels on HMBT. There is a paucity of data on the impact of SIBO in symptomatic patients after antireflux surgery to compare these findings to; however, studies have evaluated the relationship between SIBO and symptoms in patients with GERD, including those seeking antireflux surgery [11, 14, 15]. One study demonstrated that a positive methane breath test for SIBO was associated with medically refractory GERD, and among patients with medically refractory GERD, methane-positive patients had significantly more nausea (61.9% vs 35%, 0 < 0.05) with a trend toward more heartburn, regurgitation, and abdominal pain symptoms [16]. Another study found that the presence of SIBO was associated with positive symptom association probability (SAP) for reflux and regurgitation (*p* = 0.004). Additionally, patients with a positive SAP for regurgitation had more hydrogen production (275.8 ppm vs 139.1 ppm, *p* = 0.028) [14]. This study suggests that SIBO may partially drive regurgitation symptoms. Consistent with these findings, we found that treatment of SIBO not only improved gas-bloat symptoms, but also heartburn and regurgitation symptoms. Another study found that GERD patients who were SIBO-positive had significantly more bloating (74.6% vs 48.8%) and belching (60.3% vs 34.1%) than those without SIBO [11]. Similarly, we found severe gas-bloat in 77.5% of SIBO positive, but only 41.9% of SIBO-negative patients. These studies demonstrate that SIBO significantly exacerbates symptoms in patients with GERD, suggesting that SIBO may not simply co-occur with gas-bloat, it may amplify it through both physiologic and mechanical pathways after ARS.

In our cohort, 56.3% of patients with gas-bloat symptoms after ARS tested positive for SIBO. This rate mirrors previous findings in similar GERD populations, including the 60.6% prevalence observed in one cohort of 104 patients referred for antireflux surgery [11]. The incidence and prevalence of SIBO in the surgical GERD population are not well established. Furthermore, studies that report the rate of SIBO among GERD patients are limited both in sample size and design to determine true incidence and prevalence. In one study of 41 patients with typical GERD symptoms and belching, 46.3% tested positive for SIBO [14]. In another single center study 78.7% of 178 patients with GERD on at least 8 weeks of BID PPIs were found to have SIBO [16]. These high rates are consistent with what we demonstrated in our cohort of patients who underwent antireflux surgery. While robust data is necessary to establish the true impact of SIBO on GERD, these data suggest clinicians should consider SIBO testing in the management of GERD patients with gas-bloat symptoms.

Patients who’ve undergone ARS are predisposed to gas-bloat symptoms due to mechanical factors. Restoration of the LES can impair belching and reduce the ability to vent intragastric gas during gastric distention. The resulting gas accumulation is thought to impair gastric accommodation after meals, thereby exacerbating symptoms [13, 17, 18]. Even without GERD, more than two-thirds of patients with SIBO report symptoms of gas-bloat as a result of excess intraluminal gas produced through carbohydrate fermentation [19]. Therefore, from a pathophysiological standpoint, patients with both ARS and SIBO are at a significantly higher risk of developing severe gas-bloat symptoms. The fact that diagnosis and subsequent treatment of SIBO resulted in improvement of its deleterious effects, suggests that testing and treatment for SIBO, should be considered in patients who experience postoperative gas-bloat symptoms.

Treatment of SIBO led to improvement in both gas-bloat and typical reflux symptoms. Although SIBO-positive patients initially had comparable reflux but worse gas-bloat after surgery, they experienced significantly better control of both gas-bloat and reflux symptoms following treatment, compared to SIBO-negative patients. These findings suggest that SIBO represents a treatable etiology of what may otherwise have been diagnosed as gas-bloat syndrome. Recommended therapies for managing gas-bloat syndrome are largely palliative and include dietary modifications, smoking cessation, simethicone, and in some cases prokinetic drugs. Studies have previously demonstrated that patients with postoperative gas-bloat syndrome have worse GERD-HRQL scores and patient satisfaction [4]. In the present study these factors were improved by diagnosing and treating SIBO in a comparable patient population. Therefore, we recommend that SIBO testing be considered in patients with persistent postoperative gas-bloat. SIBO is not only prevalent in this population and associated with worse postoperative symptoms, but its identification also has direct therapeutic implications.

In our study, antibiotic treatment for SIBO primarily consisted of neomycin and rifaximin (77.5%), followed by cephalexin and metronidazole (12.8%). Rifaximin is the most extensively studied antibiotic for SIBO-related bloating. In a randomized controlled trial, rifaximin significantly reduced bloating scores compared to placebo, with reductions in hydrogen breath excretion strongly correlating with symptom improvement [20]. Another large clinical trial reported bloating relief in 40.2% of patients treated with rifaximin versus 30.3% in the placebo group [21]. Neomycin has also demonstrated efficacy in treating SIBO, with one study showing normalization of lactose breath tests in 75% of patients [22]. Given its responsiveness to therapy and its contribution to symptom burden, patients with persistent gas-bloat symptoms following ARS should be evaluated and treated for SIBO.

This study is not without its limitations, including its retrospective nature and modest sample size. However, it was conducted within a standardized foregut program using uniform protocols for evaluation, testing, and follow-up, which mitigates variability in clinical care. Gastroparesis is a known contributor to post-fundoplication bloating; however, studies have demonstrated good response to pyloric-directed therapy in these patients. Our aim was to evaluate patients typically managed expectantly; therefore, we excluded those with delayed gastric emptying. As a result, our findings may not be generalizable to patients with gastroparesis. Hydrogen-methane breath testing (HMBT) is a diagnostic modality with known constraints, with one meta-analysis reporting pooled sensitivity and specificity in patients who have had abdominal surgery of 81.7% and 78.8%, respectively [23]. However, HMBT remains the most practical and reproducible diagnostic tool for SIBO in routine clinical settings and, in this study, was interpreted using validated thresholds applied uniformly. Despite these limitations, the consistency of our findings, internal control comparisons, and clinically meaningful treatment effects support the validity and applicability of our conclusions.

The mechanistic factors that lead patients to develop SIBO are not well understood. One factor that may predispose patients who have undergone antireflux surgery to SIBO is their history of chronic PPI use. A consequence of acid suppression is disruption of the acid–base homeostasis in the gastrointestinal tract, which may alter the microbiome [6–10]. Many studies have demonstrated that chronic PPI use can alter the gastrointestinal microbiota and contribute to the development of SIBO [6–8, 24, 25]. One study of 1827 patients found that chronic PPI use was associated with lower diversity and higher bacterial cell counts in in the upper gastrointestinal tract, particularly Streptococci [6]. In another study, SIBO testing was performed in 200 GERD patients on PPIs, 200 irritable bowel syndrome patients and 50 healthy controls and found that SIBO was present in 50% of patients on PPIs, 24.5% of patients with IBS and 6% of healthy controls (*p* < 0.001). Additionally, there was an increase in the rate of SIBO after at least 1 year of PPI use. [8]. A meta-analysis of eleven studies (*n* = 3134) found that PPI users were 2.3 times more likely to have SIBO than non-users [26]. These findings suggest that chronic PPI use may be contributing to SIBO and symptomatology. Therefore, earlier surgical intervention may be a better option for some patients than long term medical management.

Acid suppression has long been implicated in the development of SIBO, but studies suggest it is not the sole factor [5, 16, 27–32]. In a retrospective review of 394 patients who underwent endoscopy and hydrogen-methane breath testing, the incidence of GERD was significantly higher among SIBO-positive patients compared to those who were SIBO-negative (24.4% vs 13.2%, *p* = 0.007). In multivariable analysis, GERD was the only independent predictor of SIBO (OR 1.96, *p* = 0.004), even after adjusting for PPI use [5]. These findings suggest that GERD itself may promote SIBO, potentially through an underlying motility disorder that facilitates both reflux and small intestinal bacterial overgrowth. Supporting this concept, have demonstrated significantly reduced esophageal microbiome bacterial diversity in patients with GERD patients compared to controls [33]. Together, these findings indicate that both SIBO prevalence and microbial composition are altered in GERD, likely through mechanisms beyond acid suppression [5, 34]. Although the causal relationship between GERD and SIBO remains unclear, the association is well established and may contribute to persistent foregut symptoms in some patients.

Although GERD has been shown to contribute to SIBO, emerging data suggest that SIBO may also worsen GERD. Methane-producing SIBO alters the gut microbiota, particularly Bacteroidetes, which has been associated with GERD pathogenesis [5]. Dysbiosis can lead to inflammation, impaired motility, and gas buildup, which in turn activates the vagus nerve and reduces lower esophageal sphincter (LES) pressure. Excess gas may also increase visceral sensitivity and acid exposure by widening intercellular spaces and stimulating acid-sensitive receptors. Methane slows intestinal transit and reduces circulating serotonin, further impairing motility. Additionally, SIBO may exacerbate anxiety and depression in patients with refractory GERD by disrupting the brain-gut axis, contributing to a cycle of symptom persistence [16, 32, 35]. Given the strong bidirectional relationship between SIBO and refractory GERD, it is critical to evaluate for SIBO in patients being considered for antireflux surgery. Identifying and managing SIBO may be particularly important in the postoperative setting, where its presence could contribute to persistent symptoms or compromise surgical outcomes.

## Conclusion

In this study, we identified small intestinal bacterial overgrowth (SIBO) as a clinically significant and treatable contributor to persistent gas-bloat symptoms after antireflux surgery. SIBO was present in over half of symptomatic patients and was associated with more severe bloating without differences in anatomical failure or acid suppression outcomes. Targeted antibiotic therapy led to substantial improvements in bloating severity, overall GERD-related quality of life, and patient satisfaction, surpassing outcomes in SIBO-negative patients. These findings suggest that what is often labeled as gas-bloat syndrome may, in a subset of patients, represent unrecognized SIBO. Routine testing in patients with unexplained postoperative bloating may therefore allow for therapeutic intervention and symptom resolution. Future prospective studies are warranted to validate these findings and clarify the optimal diagnostic and treatment protocols. Until then, SIBO should be considered in the differential diagnosis of gas-bloat symptoms after antireflux surgery.
